# *N*-(1-Allyl-3-chloro-4-eth­oxy-1*H*-indazol-5-yl)-4-methyl­benzene­sulfonamide

**DOI:** 10.1107/S1600536814010253

**Published:** 2014-05-10

**Authors:** Hakima Chicha, El Mostapha Rakib, Latifa Bouissane, Mohamed Saadi, Lahcen El Ammari

**Affiliations:** a Laboratoire de Chimie Organique et Analytique, Université Sultan Moulay Slimane, Faculté des Sciences et Techniques, Béni-Mellal, BP 523, Morocco; b Laboratoire de Chimie du Solide Appliquée, Faculté des Sciences, Université Mohammed V-Agdal, Avenue Ibn Battouta, BP 1014, Rabat, Morocco

## Abstract

In the title compound, C_19_H_20_ClN_3_O_3_S, the benzene ring is inclined to the indazole ring system by 51.23 (8)°. In the crystal, mol­ecules are linked by pairs of N—H⋯O hydrogen bonds, forming inversion dimers which stack in columns parallel to [011]. The atoms in the allyl group are disordered over two sets of sites with an occupancy ratio of 0.624 (8):0.376 (8).

## Related literature   

For the biological activity of sulfonamides, see: El-Sayed *et al.* (2011 ▶); Mustafa *et al.* (2012 ▶); Scozzafava *et al.* (2003 ▶); Bouissane *et al.* (2006 ▶). For similar compounds, see: Abbassi *et al.* (2012 ▶, 2013 ▶); Chicha *et al.* (2014 ▶).
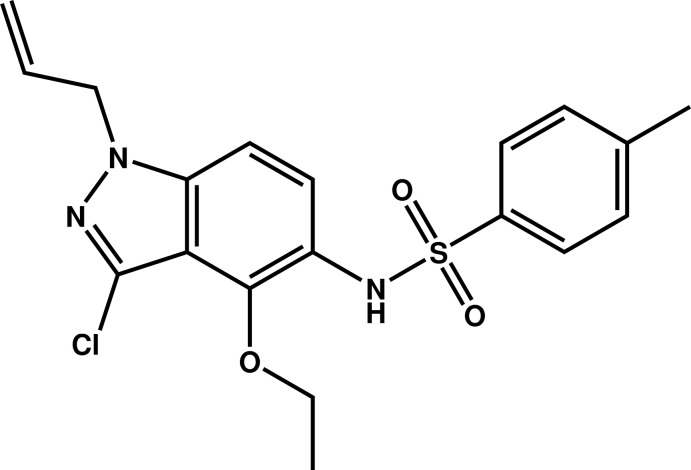


## Experimental   

### 

#### Crystal data  

C_19_H_20_ClN_3_O_3_S
*M* *_r_* = 405.89
Triclinic, 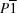

*a* = 10.0345 (2) Å
*b* = 10.5208 (2) Å
*c* = 10.7237 (2) Å
α = 71.561 (1)°
β = 69.601 (1)°
γ = 83.039 (1)°
*V* = 1006.56 (3) Å^3^
*Z* = 2
Mo *K*α radiation
μ = 0.32 mm^−1^
*T* = 296 K
0.42 × 0.35 × 0.30 mm

#### Data collection  

Bruker X8 APEX diffractometer
Absorption correction: multi-scan (*SADABS*; Bruker, 2009 ▶) *T*_min_ = 0.693, *T*_max_ = 0.747
21411 measured reflections
4792 independent reflections
3967 reflections with *I* > 2σ(*I*)
*R*_int_ = 0.027

#### Refinement  

*R*[*F* ^2^ > 2σ(*F* ^2^)] = 0.041
*wR*(*F* ^2^) = 0.126
*S* = 1.04
4792 reflections
263 parameters
H-atom parameters constrained
Δρ_max_ = 0.42 e Å^−3^
Δρ_min_ = −0.35 e Å^−3^

### 

Data collection: *APEX2* (Bruker, 2009 ▶); cell refinement: *SAINT* (Bruker, 2009 ▶); data reduction: *SAINT*; program(s) used to solve structure: *SHELXS97* (Sheldrick, 2008 ▶); program(s) used to refine structure: *SHELXL97* (Sheldrick, 2008 ▶); molecular graphics: *ORTEP-3 for Windows* (Farrugia, 2012 ▶); software used to prepare material for publication: *PLATON* (Spek, 2009 ▶) and *publCIF* (Westrip, 2010 ▶).

## Supplementary Material

Crystal structure: contains datablock(s) I. DOI: 10.1107/S1600536814010253/bt6978sup1.cif

Structure factors: contains datablock(s) I. DOI: 10.1107/S1600536814010253/bt6978Isup2.hkl

Supporting information file. DOI: 10.1107/S1600536814010253/bt6978Isup3.cml

CCDC reference: 1001396

Additional supporting information:  crystallographic information; 3D view; checkCIF report

## Figures and Tables

**Table 1:** Hydrogen-bond geometry (Å, °)

*D*—H⋯*A*	*D*—H	H⋯*A*	*D*⋯*A*	*D*—H⋯*A*
N3—H3*N*⋯O3^i^	0.89	2.22	3.0588 (17)	156

Symmetry code: (i) 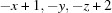.

**Fig. 1.**
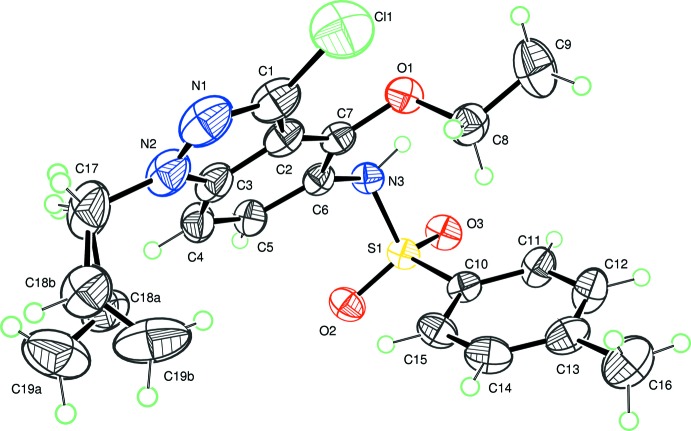
Molecular structure of the title compound with the atom-labelling scheme. Displacement ellipsoids are drawn at the 50% probability level. H atoms are represented as small circles.

**Fig. 2.**
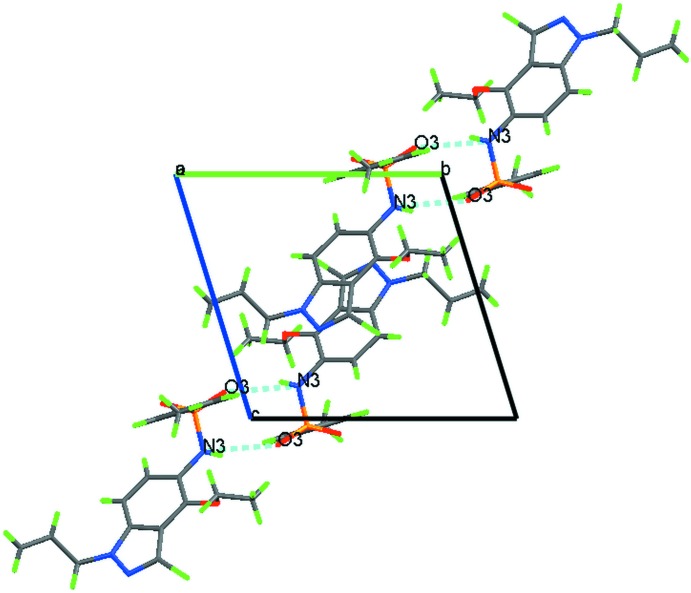
Projection of the crystal structure along a axix, showing molecules linked by hydrogen bonds and forming a dimers columns parallel to the [0 1 1] direction.

## supplementary crystallographic information

### 1. Comment

Sulfonamides are an important class of compounds which are widely used in the design of diverse classes of drug candidates (El-Sayed, *et al.*, 2011; Mustafa, *et al.*, 2012; Scozzafava, *et al.*, 2003; Bouissane, *et al.*, 2006). Previously, we identified a series of indazoles bearing a sulfonamide moiety with good antiproliferative activities. Recently, some *N*-[7(6)-indazolyl]arylsulfonamides prepared by our research group showed important antiproliferative activity against some human and murine cell lines (Abbassi, *et al.*, 2012; Abbassi, *et al.*, 2013; Chicha, *et al.*, 2014).

The molecule of the title compound is built up from two fused five- and six-membered rings (N1 N2 C1 to C7) almost coplanar, with a maximum deviation of -0.036 (2) Å for C5 atom (Fig.1). The indazol system forms a dihedral angle of 51.23 (8)° with the plane through the benzene ring and it is nearly perpendicular to the allyl group as indicated by the torsion angle C18B–C17–N2–N1 of 95.4 (5)°.

The cohesion of the crystal structure is ensured by N3–H3N···O3 hydrogen bonds between molecules forming a dimers, arranged in columns parallel to the [0 1 1] direction as shown in Fig.2 and Table 1.

### 2. Experimental

A mixture of 1-allyl-3-chloro-5-nitroindazole (1.22 mmol) and anhydrous SnCl_2_ (1.1 g, 6.1 mmol) in 25 ml of absolute ethanol was heated at 333 K for 6 h. After reduction, the starting material disappeared, and the solution was allowed to cool down. The pH was made slightly basic (pH 7–8) by addition of 5% aqueous potassium bicarbonate before extraction with ethyl acetate. The organic phase was washed with brine and dried over magnesium sulfate. The solvent was removed to afford the amine, which was immediately dissolved in pyridine (5 ml) and then reacted with 4-methylbenzenesulfonyl chloride (1.25 mmol) at room temperature for 24 h. After the reaction mixture was concentrated *in vacuo*, the resulting residue was purified by flash chromatography (eluted with Ethyl acetate: Hexane 2:8). The title compound was recrystallized from ethanol (yield = 40%, m.p. = 373 K).

### 3. Refinement

The reflections (100), (010), (001) and (011) are removed from the refinement because they are affected by the beam stop. The structure is solved by direct method technique and refined by full-matrix least-squares using *SHELXS97* and *SHELXL97* program packages. H atoms were located in a difference map and treated as riding with C–H = 0.96 Å, C–H = 0.97 Å, C–H = 0.93 Å, and N–H = 0.89 Å for methyl, methylene, aromatic CH, and NH, respectively. All hydrogen with *U*_iso_(H) = 1.2 *U*_eq_ (aromatic, methylene, NH)and *U*_iso_(H) = 1.5 *U*_eq_ for methyl.

### Crystal data

**Table:**

C_19_H_20_ClN_3_O_3_S	*Z* = 2
*M**_r_* = 405.89	*F*(000) = 424
Triclinic, *P*1	*D*_x_ = 1.339 Mg m^−^^3^
Hall symbol: -P 1	Melting point: 373 K
*a* = 10.0345 (2) Å	Mo *K*α radiation, λ = 0.71073 Å
*b* = 10.5208 (2) Å	Cell parameters from 4792 reflections
*c* = 10.7237 (2) Å	θ = 2.5–27.9°
α = 71.561 (1)°	µ = 0.32 mm^−^^1^
β = 69.601 (1)°	*T* = 296 K
γ = 83.039 (1)°	Block, colourless
*V* = 1006.56 (3) Å^3^	0.42 × 0.35 × 0.30 mm

### Data collection

**Table:**

Bruker X8 APEX diffractometer	4792 independent reflections
Radiation source: fine-focus sealed tube	3967 reflections with *I* > 2σ(*I*)
Graphite monochromator	*R*_int_ = 0.027
φ and ω scans	θ_max_ = 27.9°, θ_min_ = 2.5°
Absorption correction: multi-scan (*SADABS*; Bruker, 2009)	*h* = −13→13
*T*_min_ = 0.693, *T*_max_ = 0.747	*k* = −13→13
21411 measured reflections	*l* = −14→13

### Refinement

**Table:**

Refinement on *F*^2^	Primary atom site location: structure-invariant direct methods
Least-squares matrix: full	Secondary atom site location: difference Fourier map
*R*[*F*^2^ > 2σ(*F*^2^)] = 0.041	Hydrogen site location: inferred from neighbouring sites
*wR*(*F*^2^) = 0.126	H-atom parameters constrained
*S* = 1.04	*w* = 1/[σ^2^(*F*_o_^2^) + (0.0687*P*)^2^ + 0.2408*P*] where *P* = (*F*_o_^2^ + 2*F*_c_^2^)/3
4792 reflections	(Δ/σ)_max_ = 0.001
263 parameters	Δρ_max_ = 0.42 e Å^−^^3^
0 restraints	Δρ_min_ = −0.35 e Å^−^^3^

### Special details

**Table:**

Geometry. All e.s.d.'s (except the e.s.d. in the dihedral angle between two l.s. planes) are estimated using the full covariance matrix. The cell e.s.d.'s are taken into account individually in the estimation of e.s.d.'s in distances, angles and torsion angles; correlations between e.s.d.'s in cell parameters are only used when they are defined by crystal symmetry. An approximate (isotropic) treatment of cell e.s.d.'s is used for estimating e.s.d.'s involving l.s. planes.
Refinement. Refinement of *F*^2^ against all reflections. The weighted *R*-factor *wR* and goodness of fit *S* are based on *F*^2^, conventional *R*-factors *R* are based on *F*, with *F* set to zero for negative *F*^2^. The threshold expression of *F*^2^ > σ(*F*^2^) is used only for calculating *R*-factors(gt) *etc*. and is not relevant to the choice of reflections for refinement. *R*-factors based on *F*^2^ are statistically about twice as large as those based on *F*, and *R*- factors based on all data will be even larger.

### Fractional atomic coordinates and isotropic or equivalent isotropic displacement parameters (Å^2^)

**Table:**

	*x*	*y*	*z*	*U*_iso_*/*U*_eq_	Occ. (<1)
C1	0.8128 (2)	0.5118 (2)	0.42585 (19)	0.0628 (5)	
C2	0.69725 (19)	0.46383 (17)	0.55054 (17)	0.0484 (4)	
C3	0.6105 (2)	0.57806 (17)	0.55830 (18)	0.0526 (4)	
C4	0.4795 (2)	0.57522 (17)	0.6626 (2)	0.0544 (4)	
H4	0.4230	0.6519	0.6653	0.065*	
C5	0.43792 (18)	0.45479 (17)	0.76086 (18)	0.0481 (4)	
H5	0.3495	0.4488	0.8299	0.058*	
C6	0.52561 (16)	0.33930 (15)	0.76030 (16)	0.0393 (3)	
C7	0.65446 (17)	0.34152 (16)	0.65574 (16)	0.0415 (3)	
C8	0.8599 (2)	0.2160 (2)	0.6873 (2)	0.0608 (5)	
H8A	0.8369	0.2187	0.7821	0.073*	
H8B	0.9213	0.2907	0.6252	0.073*	
C9	0.9321 (3)	0.0880 (3)	0.6722 (4)	0.0976 (9)	
H9A	0.9546	0.0866	0.5780	0.146*	
H9B	0.8705	0.0149	0.7343	0.146*	
H9C	1.0181	0.0796	0.6945	0.146*	
C10	0.62203 (17)	0.22040 (15)	1.03861 (15)	0.0404 (3)	
C11	0.7030 (2)	0.10747 (17)	1.0730 (2)	0.0542 (4)	
H11	0.6664	0.0226	1.0973	0.065*	
C12	0.8389 (2)	0.1218 (2)	1.0708 (2)	0.0618 (5)	
H12	0.8932	0.0457	1.0949	0.074*	
C13	0.8960 (2)	0.2475 (2)	1.0333 (2)	0.0570 (4)	
C14	0.8130 (2)	0.35895 (19)	0.9984 (2)	0.0586 (5)	
H14	0.8502	0.4438	0.9724	0.070*	
C15	0.67618 (19)	0.34768 (16)	1.00100 (19)	0.0510 (4)	
H15	0.6214	0.4237	0.9781	0.061*	
C16	1.0466 (2)	0.2623 (3)	1.0261 (3)	0.0839 (7)	
H16A	1.0877	0.1752	1.0543	0.126*	
H16B	1.0460	0.3125	1.0870	0.126*	
H16C	1.1017	0.3086	0.9323	0.126*	
C17	0.6318 (3)	0.8191 (2)	0.4084 (3)	0.0869 (8)	
H17A	0.5322	0.8251	0.4162	0.104*	0.376 (8)
H17B	0.6870	0.8653	0.3133	0.104*	0.376 (8)
H17C	0.6737	0.8565	0.3084	0.104*	0.624 (8)
H17D	0.5295	0.8209	0.4297	0.104*	0.624 (8)
C18A	0.657 (2)	0.8881 (16)	0.5179 (15)	0.082 (4)	0.376 (8)
H18A	0.6423	0.8383	0.6103	0.099*	0.376 (8)
C19A	0.6994 (8)	1.0147 (9)	0.4734 (11)	0.105 (4)	0.376 (8)
H19A	0.7144	1.0645	0.3810	0.126*	0.376 (8)
H19B	0.7136	1.0533	0.5346	0.126*	0.376 (8)
C18B	0.6631 (11)	0.9034 (11)	0.4701 (8)	0.076 (2)	0.624 (8)
H18B	0.6512	0.9951	0.4335	0.091*	0.624 (8)
C19B	0.7085 (7)	0.8617 (7)	0.5769 (6)	0.109 (2)	0.624 (8)
H19C	0.7214	0.7705	0.6156	0.131*	0.624 (8)
H19D	0.7279	0.9232	0.6141	0.131*	0.624 (8)
N1	0.8009 (2)	0.6396 (2)	0.36458 (18)	0.0738 (5)	
N2	0.6767 (2)	0.68077 (16)	0.44501 (18)	0.0663 (5)	
N3	0.47799 (13)	0.21562 (12)	0.86652 (13)	0.0403 (3)	
H3N	0.5266	0.1437	0.8473	0.048*	
O1	0.73089 (12)	0.22498 (12)	0.65275 (13)	0.0491 (3)	
O2	0.36643 (13)	0.31502 (12)	1.06165 (13)	0.0531 (3)	
O3	0.40499 (13)	0.07043 (11)	1.10772 (12)	0.0510 (3)	
S1	0.45343 (4)	0.20409 (3)	1.03010 (4)	0.04001 (12)	
Cl1	0.95478 (8)	0.42370 (8)	0.34929 (7)	0.1012 (3)	

### Atomic displacement parameters (Å^2^)

**Table:**

	*U*^11^	*U*^22^	*U*^33^	*U*^12^	*U*^13^	*U*^23^
C1	0.0652 (12)	0.0749 (13)	0.0401 (9)	−0.0231 (10)	−0.0109 (8)	−0.0048 (8)
C2	0.0515 (9)	0.0554 (9)	0.0379 (8)	−0.0137 (7)	−0.0157 (7)	−0.0071 (7)
C3	0.0673 (11)	0.0476 (9)	0.0439 (9)	−0.0139 (8)	−0.0265 (8)	−0.0008 (7)
C4	0.0632 (11)	0.0446 (9)	0.0567 (10)	0.0020 (8)	−0.0281 (9)	−0.0083 (7)
C5	0.0456 (8)	0.0487 (9)	0.0488 (9)	−0.0008 (7)	−0.0168 (7)	−0.0110 (7)
C6	0.0407 (7)	0.0400 (7)	0.0384 (7)	−0.0078 (6)	−0.0157 (6)	−0.0073 (6)
C7	0.0425 (8)	0.0457 (8)	0.0391 (8)	−0.0063 (6)	−0.0154 (6)	−0.0119 (6)
C8	0.0471 (9)	0.0792 (13)	0.0574 (11)	0.0035 (9)	−0.0184 (8)	−0.0219 (10)
C9	0.0637 (14)	0.0928 (19)	0.130 (3)	0.0210 (13)	−0.0312 (15)	−0.0325 (17)
C10	0.0447 (8)	0.0391 (7)	0.0360 (7)	−0.0108 (6)	−0.0111 (6)	−0.0079 (6)
C11	0.0628 (11)	0.0399 (8)	0.0589 (10)	−0.0141 (7)	−0.0297 (9)	0.0024 (7)
C12	0.0617 (11)	0.0572 (10)	0.0675 (12)	−0.0071 (9)	−0.0349 (10)	−0.0020 (9)
C13	0.0535 (10)	0.0704 (12)	0.0509 (10)	−0.0184 (9)	−0.0165 (8)	−0.0172 (9)
C14	0.0591 (10)	0.0522 (10)	0.0657 (12)	−0.0222 (8)	−0.0117 (9)	−0.0211 (8)
C15	0.0527 (9)	0.0395 (8)	0.0596 (10)	−0.0088 (7)	−0.0118 (8)	−0.0176 (7)
C16	0.0610 (13)	0.1033 (18)	0.0955 (18)	−0.0223 (12)	−0.0326 (13)	−0.0248 (15)
C17	0.120 (2)	0.0525 (11)	0.0877 (17)	−0.0236 (12)	−0.0575 (16)	0.0148 (11)
C18A	0.134 (10)	0.060 (6)	0.078 (9)	−0.010 (5)	−0.065 (9)	−0.018 (6)
C19A	0.084 (5)	0.107 (7)	0.159 (9)	0.003 (4)	−0.044 (5)	−0.083 (6)
C18B	0.081 (3)	0.071 (3)	0.067 (4)	−0.021 (2)	−0.022 (3)	−0.004 (3)
C19B	0.121 (4)	0.136 (5)	0.081 (4)	−0.061 (4)	−0.011 (3)	−0.048 (3)
N1	0.0861 (13)	0.0787 (12)	0.0463 (9)	−0.0342 (10)	−0.0185 (9)	0.0039 (8)
N2	0.0899 (13)	0.0540 (9)	0.0496 (9)	−0.0224 (8)	−0.0283 (9)	0.0058 (7)
N3	0.0415 (7)	0.0359 (6)	0.0420 (7)	−0.0079 (5)	−0.0112 (5)	−0.0095 (5)
O1	0.0445 (6)	0.0508 (6)	0.0524 (7)	−0.0024 (5)	−0.0131 (5)	−0.0185 (5)
O2	0.0518 (7)	0.0486 (6)	0.0524 (7)	−0.0007 (5)	−0.0070 (5)	−0.0173 (5)
O3	0.0513 (7)	0.0411 (6)	0.0489 (7)	−0.0169 (5)	−0.0053 (5)	−0.0039 (5)
S1	0.0401 (2)	0.0354 (2)	0.0385 (2)	−0.00946 (14)	−0.00577 (15)	−0.00744 (14)
Cl1	0.0849 (4)	0.1221 (6)	0.0606 (4)	−0.0129 (4)	0.0153 (3)	−0.0182 (4)

### Geometric parameters (Å, º)

**Table:**

C1—N1	1.308 (3)	C13—C14	1.383 (3)
C1—C2	1.422 (3)	C13—C16	1.510 (3)
C1—Cl1	1.705 (2)	C14—C15	1.382 (3)
C2—C3	1.403 (3)	C14—H14	0.9300
C2—C7	1.411 (2)	C15—H15	0.9300
C3—N2	1.366 (2)	C16—H16A	0.9600
C3—C4	1.394 (3)	C16—H16B	0.9600
C4—C5	1.366 (2)	C16—H16C	0.9600
C4—H4	0.9300	C17—C18B	1.380 (11)
C5—C6	1.412 (2)	C17—N2	1.442 (3)
C5—H5	0.9300	C17—C18A	1.663 (15)
C6—C7	1.382 (2)	C17—H17A	0.9700
C6—N3	1.4327 (18)	C17—H17B	0.9700
C7—O1	1.3683 (19)	C17—H17C	0.9700
C8—O1	1.450 (2)	C17—H17D	0.9700
C8—C9	1.478 (3)	C18A—C19A	1.331 (19)
C8—H8A	0.9700	C18A—H18A	0.9300
C8—H8B	0.9700	C19A—H19A	0.9300
C9—H9A	0.9600	C19A—H19B	0.9300
C9—H9B	0.9600	C18B—C19B	1.307 (10)
C9—H9C	0.9600	C18B—H18B	0.9300
C10—C11	1.380 (2)	C19B—H19C	0.9300
C10—C15	1.390 (2)	C19B—H19D	0.9300
C10—S1	1.7572 (16)	N1—N2	1.357 (3)
C11—C12	1.382 (3)	N3—S1	1.6504 (13)
C11—H11	0.9300	N3—H3N	0.8896
C12—C13	1.388 (3)	O2—S1	1.4267 (12)
C12—H12	0.9300	O3—S1	1.4317 (11)
			
N1—C1—C2	112.7 (2)	C13—C16—H16B	109.5
N1—C1—Cl1	118.98 (16)	H16A—C16—H16B	109.5
C2—C1—Cl1	128.32 (17)	C13—C16—H16C	109.5
C3—C2—C7	119.20 (16)	H16A—C16—H16C	109.5
C3—C2—C1	103.15 (16)	H16B—C16—H16C	109.5
C7—C2—C1	137.65 (18)	C18B—C17—N2	118.3 (5)
N2—C3—C4	130.22 (18)	C18B—C17—C18A	14.3 (7)
N2—C3—C2	106.89 (17)	N2—C17—C18A	107.4 (6)
C4—C3—C2	122.81 (15)	C18B—C17—H17A	111.6
C5—C4—C3	117.00 (16)	N2—C17—H17A	110.2
C5—C4—H4	121.5	C18A—C17—H17A	110.2
C3—C4—H4	121.5	C18B—C17—H17B	96.9
C4—C5—C6	121.71 (16)	N2—C17—H17B	110.2
C4—C5—H5	119.1	C18A—C17—H17B	110.2
C6—C5—H5	119.1	H17A—C17—H17B	108.5
C7—C6—C5	121.27 (14)	C18B—C17—H17C	107.7
C7—C6—N3	119.03 (14)	N2—C17—H17C	107.7
C5—C6—N3	119.65 (14)	C18A—C17—H17C	121.4
O1—C7—C6	119.44 (14)	H17A—C17—H17C	99.4
O1—C7—C2	122.54 (14)	H17B—C17—H17C	11.9
C6—C7—C2	117.89 (15)	C18B—C17—H17D	107.7
O1—C8—C9	107.74 (18)	N2—C17—H17D	107.7
O1—C8—H8A	110.2	C18A—C17—H17D	104.8
C9—C8—H8A	110.2	H17A—C17—H17D	7.7
O1—C8—H8B	110.2	H17B—C17—H17D	116.0
C9—C8—H8B	110.2	H17C—C17—H17D	107.1
H8A—C8—H8B	108.5	C19A—C18A—C17	120.0 (9)
C8—C9—H9A	109.5	C19A—C18A—H18A	120.0
C8—C9—H9B	109.5	C17—C18A—H18A	120.0
H9A—C9—H9B	109.5	C18A—C19A—H19A	120.0
C8—C9—H9C	109.5	C18A—C19A—H19B	120.0
H9A—C9—H9C	109.5	H19A—C19A—H19B	120.0
H9B—C9—H9C	109.5	C19B—C18B—C17	123.8 (9)
C11—C10—C15	120.75 (15)	C19B—C18B—H18B	118.1
C11—C10—S1	119.82 (11)	C17—C18B—H18B	118.1
C15—C10—S1	119.27 (13)	C18B—C19B—H19C	120.0
C10—C11—C12	119.32 (15)	C18B—C19B—H19D	120.0
C10—C11—H11	120.3	H19C—C19B—H19D	120.0
C12—C11—H11	120.3	C1—N1—N2	105.77 (17)
C11—C12—C13	121.25 (18)	N1—N2—C3	111.52 (17)
C11—C12—H12	119.4	N1—N2—C17	120.35 (19)
C13—C12—H12	119.4	C3—N2—C17	128.1 (2)
C14—C13—C12	118.22 (17)	C6—N3—S1	119.49 (10)
C14—C13—C16	120.82 (18)	C6—N3—H3N	114.3
C12—C13—C16	120.9 (2)	S1—N3—H3N	108.7
C15—C14—C13	121.79 (16)	C7—O1—C8	114.94 (13)
C15—C14—H14	119.1	O2—S1—O3	119.60 (7)
C13—C14—H14	119.1	O2—S1—N3	107.72 (7)
C14—C15—C10	118.65 (16)	O3—S1—N3	105.18 (7)
C14—C15—H15	120.7	O2—S1—C10	108.63 (7)
C10—C15—H15	120.7	O3—S1—C10	108.69 (7)
C13—C16—H16A	109.5	N3—S1—C10	106.23 (7)

### Hydrogen-bond geometry (Å, º)

**Table:**

*D*—H···*A*	*D*—H	H···*A*	*D*···*A*	*D*—H···*A*
N3—H3*N*···O3^i^	0.89	2.22	3.0588 (17)	156

Symmetry code: (i) −*x*+1, −*y*, −*z*+2.
